# Fatigue Life Prediction of CFRP-FBG Sensor-Reinforced RC Beams Enabled by LSTM-Based Deep Learning

**DOI:** 10.3390/polym17152112

**Published:** 2025-07-31

**Authors:** Minrui Jia, Chenxia Zhou, Xiaoyuan Pei, Zhiwei Xu, Wen Xu, Zhenkai Wan

**Affiliations:** 1 Engineering Teaching Practice Training Center of Tiangong University, Tianjin 300072, China; jiaminrui@tiangong.edu.cn (M.J.); wanzhenkai@tiangong.edu.cn (Z.W.); 2 Key Laboratory of Advanced Braided Composites, Ministry of Education, School of Textile Science and Engineering, Tiangong University, Tianjin 300387, China; zcx191226@foxmail.com (C.Z.); xuzhiwei@tiangong.edu.cn (Z.X.); 3 Carbon Technology Group Co., Ltd., Tianjin 300380, China; xwtj95@163.com

**Keywords:** LSTM neural network, CFRP-FBG sensors, fatigue life prediction, principal component analysis, deep learning

## Abstract

Amidst the escalating demand for high-precision structural health monitoring in large-scale engineering applications, carbon fiber-reinforced polymer fiber Bragg grating (CFRP-FBG) sensors have emerged as a pivotal technology for fatigue life evaluation, owing to their exceptional sensitivity and intrinsic immunity to electromagnetic interference. A time-series predictive architecture based on long short-term memory (LSTM) networks is developed in this work to facilitate intelligent fatigue life assessment of structures subjected to complex cyclic loading by capturing and modeling critical spectral characteristics of CFRP-FBG sensors, specifically the side-mode suppression ratio and main-lobe peak-to-valley ratio. To enhance model robustness and generalization, Principal Component Analysis (PCA) was employed to isolate the most salient spectral features, followed by data preprocessing via normalization and model optimization through the integration of the Adam optimizer and Dropout regularization strategy. Relative to conventional Backpropagation (BP) neural networks, the LSTM model demonstrated a substantial improvement in predicting the side-mode suppression ratio, achieving a 61.62% reduction in mean squared error (MSE) and a 34.99% decrease in root mean squared error (RMSE), thereby markedly enhancing robustness to outliers and ensuring greater overall prediction stability. In predicting the peak-to-valley ratio, the model attained a notable 24.9% decrease in mean absolute error (MAE) and a 21.2% reduction in root mean squared error (RMSE), thereby substantially curtailing localized inaccuracies. The forecasted confidence intervals were correspondingly narrower and exhibited diminished fluctuation, highlighting the LSTM architecture’s enhanced proficiency in capturing nonlinear dynamics and modeling temporal dependencies. The proposed method manifests considerable practical engineering relevance and delivers resilient intelligent assistance for the seamless implementation of CFRP-FBG sensor technology in structural health monitoring and fatigue life prognostics.

## 1. Introduction

With the progressive aging of infrastructure systems, fatigue-induced deterioration resulting from cyclic mechanical loading, environmental corrosion, and thermal fluctuations has emerged as a critical challenge, significantly undermining structural reliability and long-term serviceability [1,2,3]. As the foundational elements of contemporary civil infrastructure, reinforced concrete (RC) structures are extensively deployed in strategic applications such as bridge decks [4], tunnel linings [5], and high-rise structural cores [6]. Under sustained fatigue conditions, these systems are prone to complex degradation phenomena, including microcrack nucleation and propagation in the concrete matrix, plastic yielding of steel reinforcement, and progressive deterioration of the concrete cover. These coupled mechanisms collectively compromise structural integrity and accelerate functional obsolescence, thereby attracting heightened scrutiny from both researchers and practitioners in structural engineering domains [7].

In recent years, CFRP has garnered significant attention as a high-performance material for the rehabilitation and strengthening of RC structures, owing to its exceptional specific strength, superior corrosion resistance, and ease of on-site deployment [8,9]. For instance, the strategic application of externally bonded CFRP laminates has been empirically validated to markedly extend the fatigue life of structural elements in highway bridges [10]. Similarly, in rail transit infrastructure, CFRP has demonstrated notable efficacy in retarding crack propagation and mitigating fatigue-induced failure mechanisms [11]. Concurrently, FBG sensors have emerged as a pivotal technology in the realm of Structural Health Monitoring (SHM) due to their intrinsic advantages such as ultra-high sensitivity, robust immunity to electromagnetic interference, multiplexing capability, and compact geometry [12,13,14]. The synergistic integration of CFRP reinforcement with FBG sensing facilitates the realization of multifunctional “reinforcement–monitoring integrated” systems, enabling in situ, real-time tracking of structural fatigue damage and quantitative characterization of degradation dynamics. This integrated paradigm offers considerable potential for deployment in mission-critical assets such as wind turbine blades, aerospace components, and railway box girders [15,16,17].

Nevertheless, the harsh and variable operational environments encountered by CFRP-FBG composite systems often result in signal responses exhibiting pronounced nonlinearity, nonstationarity, and long-term temporal dependencies. These characteristics pose significant challenges for traditional modeling techniques—particularly linear regression and shallow neural networks such as the Backpropagation Neural Network (BPNN)—which lack the capacity to accurately capture complex spatiotemporal patterns and exhibit poor robustness and generalization when extrapolating degradation trajectories. Hence, there exists a compelling need for advanced data-driven models with superior sequence learning capabilities to facilitate accurate and reliable fatigue life prediction.

Deep learning architectures, particularly Recurrent Neural Networks (RNNs) and their advanced derivatives, have shown exceptional promise in modeling nonlinear, high-dimensional time-series data with long-range dependencies [18]. Among these, the LSTM network—characterized by its gated memory cell structure encompassing input, forget, and output gates—effectively overcomes the vanishing gradient problem endemic to conventional RNNs. LSTM has been widely and successfully deployed across various domains including machinery prognostics, composite material fatigue modeling, and structural degradation forecasting [19].

This study presents a fatigue life prediction approach for CFRP-FBG sensors subjected to multi-cycle fatigue loading, leveraging the capabilities of Long Short-Term Memory (LSTM) neural networks. Key spectral response features were extracted from the sensor signals, and dimensionality reduction was performed using Principal Component Analysis (PCA), followed by min–max normalization to enhance training efficiency and model generalization. A multi-layer LSTM architecture was developed, integrating Dropout regularization to mitigate overfitting and the Adam optimizer for adaptive learning rate adjustment. The proposed model was applied to predict the long-term evolution of fatigue-sensitive indicators, specifically the side-mode suppression ratio and main-lobe peak-to-valley ratio. Compared to a conventional Backpropagation (BP) neural network, the LSTM model exhibited significantly improved predictive accuracy and robustness, especially in capturing nonlinear degradation patterns and long-range temporal dependencies. These results demonstrate the LSTM network’s effectiveness as a data-driven tool for reliable lifetime estimation of smart optical sensors under complex fatigue conditions.

## 2. Experimental Methodology and Data Acquisition

### 2.1. Structural Configuration of CFRP-FBG Sensors

The CFRP-FBG sensor utilized in this study comprises fiber Bragg grating (FBG) sensors embedded within multiple carbon fiber bundles. These FBG sensors are internally encapsulated by the carbon fiber tows and subsequently subjected to pultrusion with resin to achieve an integrated composite structure. This manufacturing approach significantly enhances the efficiency of strain transfer and substantially improves the sensor’s fatigue durability and long-term operational stability. The carbon fibers selected for this study were SYT45S high-strength carbon fibers (24K specification, Grade A), manufactured by Zhongfu Shenying Carbon Fiber Co., Ltd., Xining, Qinghai, China. A total of 160 bundles were utilized, each containing approximately 24,000 individual filaments. The pultrusion resin system was procured from Carbon Technology Group Co., Ltd., Nanjing, Jiangsu, China comprising two components, designated as A and B, which were thoroughly mixed at a mass ratio of 5:4 prior to application. The comprehensive performance specifications of the FBG sensors are detailed in Table 1.

### 2.2. Specimen Preparation

**Figure 1:**
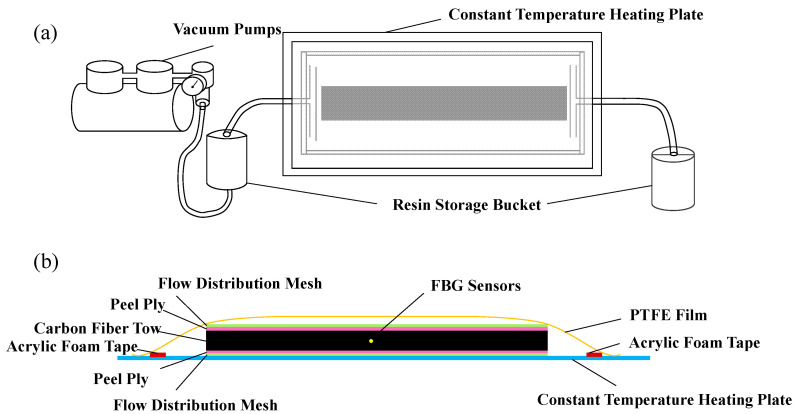
(**a**) Top-view schematic of CFRP-FBG sensor fabrication. (**b**) Side-view schematic of CFRP-FBG sensor fabrication.

The specimens investigated in this study were reinforced concrete (RC) beams externally strengthened with CFRP-FBG sensor assemblies. Each specimen consisted of two main components: the CFRP-FBG sensor unit and the RC beam substrate, which served as the structural base. The CFRP-FBG sensor comprises multiple carbon fiber bundles enveloping fiber Bragg gratings (FBGs), integrally encapsulated via resin pultrusion to form a robust and stable strain transfer system. Figure 1 presents schematic top and side views of the CFRP-FBG sensor fabrication process.

**Figure 2:**
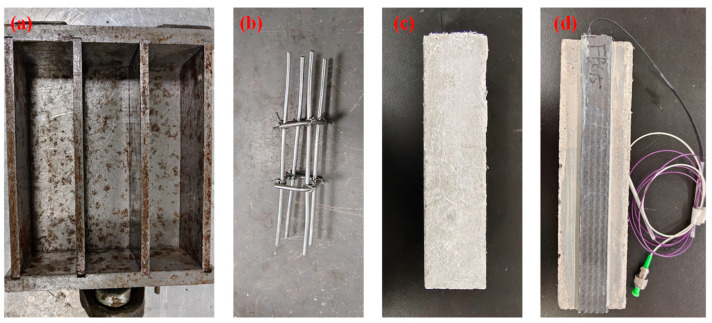
(**a**) Experimental apparatus for casting reinforced concrete specimens. (**b**) Schematic of the steel stirrups. (**c**) Three-point bending specimen of the RC beam (**d**) RC beam specimen reinforced with CFRP-FBG sensor.

The RC beam specimens were fabricated using steel stirrups measuring 140 × 20 × 20 mm, embedded within a cement mortar matrix with a mortar-to-water mass ratio of 100:17. Following casting, the specimens underwent ambient temperature curing for 24 h, followed by water curing for 28 days to ensure mechanical stability and performance consistency. The specimen preparation setup and procedures are illustrated in Figure 2a–c. All specimens were prepared according to standardized procedures and relevant technical specifications. To ensure the reliability of the preparation process, the fabricated specimens were compared against standardized reference samples. The results revealed no significant differences in mechanical properties, thereby confirming the consistency and reproducibility of the fabrication method.

The finalized specimens were assembled by externally bonding the CFRP-FBG sensors to the surface of the RC beams using an epoxy adhesive. The bonding agent consisted of components A and B of carbon plate epoxy resin mixed at a mass ratio of 2:1, then evenly applied to the bonding interface. The specimens were cured at ambient temperature for 7 days, forming an integrated reinforcement system, as shown in Figure 2d.

### 2.3. Dynamic Cyclic Loading Experiments and Sensor Data Collection

**Figure 3:**
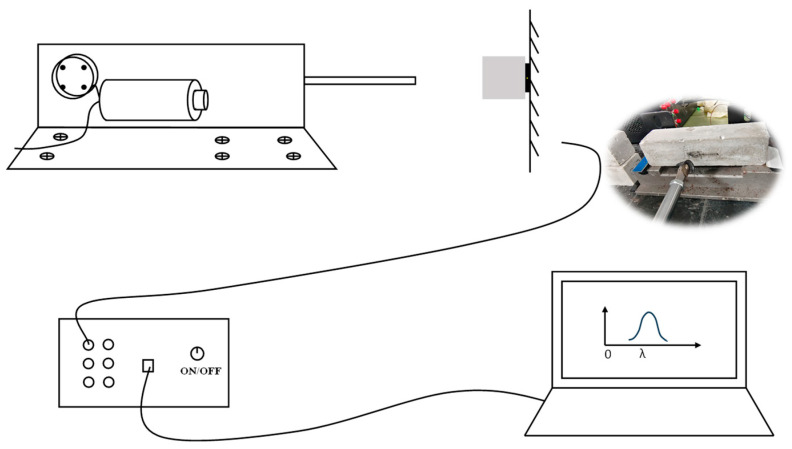
Schematic illustration of the custom-designed dynamic cyclic loading apparatus used for fatigue testing.

To simulate the fatigue damage evolution of CFRP-FBG sensor-reinforced RC beams under long-term service conditions, a custom-designed dynamic cyclic loading apparatus was employed to conduct staged fatigue loading tests on the specimens. The number of fatigue cycles was sequentially set at 200,000; 300,000; 400,000; 500,000; 1,000,000; and 1,650,000, thereby covering the entire degradation process from early-stage damage to advanced deterioration. The loading amplitude was fixed at 2% of the static failure load of the reinforced RC beam, and the loading frequency was maintained at 100 cycles per minute to ensure that the applied fatigue stress remained within a stable low-strain regime. Throughout the loading process, an FBG demodulation system was employed to continuously monitor and record the spectral response of the specimens at various fatigue stages. Data acquisition was conducted at fixed intervals of every 10,000 loading cycles, ensuring a comprehensive and high-resolution dataset capturing the full progression of fatigue-induced damage. A schematic overview of the experimental procedure is provided in Figure 3.

The spectral data acquired from the FBG sensors were systematically processed using the MATLAB 8.0 Natick, Massachusetts, USA platform to establish a wavelength–power spectral database, thereby providing high-quality raw data support for subsequent fatigue life modeling based on the LSTM neural network. For each fatigue loading stage, a complete set of wavelength–power response curves was recorded. The data were formatted as two-dimensional spectral functions (λ–P), representing the functional relationship between the optical signal wavelength (λ) and the corresponding reflected power (P).

To comprehensively extract fatigue-sensitive features from the spectral responses, MATLAB programming was employed to analyze and quantify each set of spectral data. Eight key characteristic parameters were extracted: main peak wavelength, main peak power, main peak valley, side peak wavelength, side peak power, side mode suppression ratio, main lobe peak-to-valley ratio, and 3 dB bandwidth. These parameters collectively capture the evolution of the spectral profile across different fatigue stages, facilitating a deeper understanding of the intrinsic relationship between sensor responses and the structural fatigue condition.

**Figure 4:**
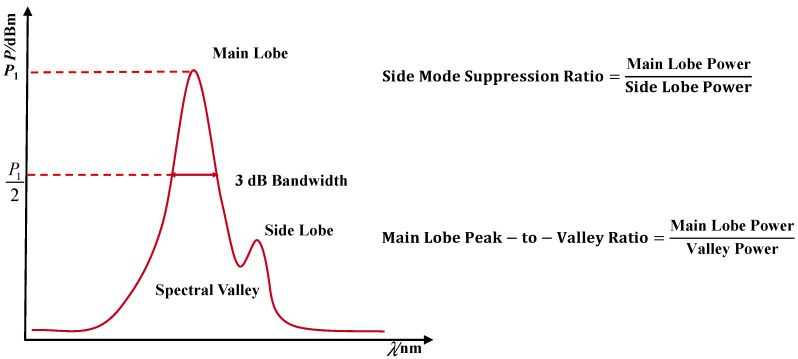
Definitions of key spectral parameters.

As illustrated in Figure 4, the definitions and physical significances of each parameter are as follows.

Main Peak Wavelength: The wavelength corresponding to the maximum reflection intensity in the spectrum, representing the current central strain value measured by the FBG sensor. Its shift during the loading process reflects the overall structural stress variation.

Main Peak Power: The maximum reflected power at the main peak position, indicating the concentration of signal energy, which is influenced by strain transfer efficiency and interface damage.

Main Peak Valley: The minimum power point adjacent to the main peak, often used to evaluate the smoothness of the main peak boundary transition, reflecting interference noise or spectral distortion.

Side Peak Wavelength and Side Peak Power: Local extrema outside the main peak, typically caused by side modes, serving as important indicators for spectral integrity and system crosstalk suppression capability.

Side Mode Suppression Ratio: Defined as the ratio of the main peak’s maximum power to the maximum power of the side peaks, reflecting the energy contrast between the main peak and side modes. A higher SMSR indicates better main peak signal-to-noise ratio and stronger system stability. A decrease in SMSR often signals the emergence of microcracks, interface degradation, or disturbed strain transfer paths within the structure, resulting in enhanced side peaks.

Main Lobe Peak-to-Valley Ratio: Defined as the ratio between the maximum power of the main peak and its adjacent valley power, characterizing the clarity and boundary sharpness of the main peak. A lower PVR suggests peak blunting, possibly caused by grating damage, interface debonding, or optical signal attenuation.

3 dB Bandwidth: The wavelength interval over which the main peak power drops to half of its maximum value, reflecting the spectral width expansion of the main peak. An increase in 3 dB bandwidth generally indicates heightened strain field non-uniformity or spectral broadening due to damage evolution, demonstrating strong sensitivity to fatigue.

By continuously extracting and temporally tracking the aforementioned parameters, the spectral evolution characteristics of the FBG sensor under fatigue loading can be systematically characterized. This approach provides both theoretical support and a robust data foundation for constructing a high-dimensional input feature set tailored for fatigue life prediction.

## 3. Feature Extraction and Data Preprocessing

### 3.1. Dimensionality Reduction in Feature Space

In the aforementioned dynamic cyclic fatigue loading experiments, eight damage-sensitive parameters were extracted. Nevertheless, pronounced multicollinearity among these parameters engenders substantial information redundancy. To facilitate a more intuitive and comprehensive characterization of the structural damage evolution throughout the fatigue lifecycle, it is imperative to attenuate the inter-parameter dependencies, thereby enabling a parsimonious representation that captures maximal damage-relevant information with minimal dimensionality.

Principal Component Analysis (PCA) is a commonly employed dimensionality reduction technique for preprocessing high-dimensional data. The core concept involves applying a linear transformation to convert the original correlated variables into a set of orthogonal principal components that retain the maximum possible variance of the original dataset. This dimensionality reduction not only eliminates noise and redundant information but also enhances computational efficiency and improves the interpretability of damage-sensitive features.

This study utilizes PCA for dimensionality reduction, following these primary steps:

(1) Standardization of the original dataset.
(2) Calculation of the correlation matrix R among variables.
(3) Determination of eigenvalues and eigenvectors of the correlation matrix R.
(4) Construction of principal components.
(5) Computation of composite scores.

Let *x*_1_, *x*_2_, …, *x_p_* represent the *p* damage detection parameters, and *c*_1_, *c*_2_, …, *c_p_* denote the corresponding weights. Each principal component is defined as a weighted linear combination of the original variables: *s* = *c*_1_*x*_1_ + *c*_2_*x*_2_ + … + *c_p_x_p_*

Generally, a single principal component cannot fully represent all the information contained in the original variables. Therefore, additional principal components are extracted sequentially. Each subsequent principal component is orthogonal to the preceding ones, meaning their covariance is zero, which ensures the statistical independence of the components.

(1) Standardization of the original dataset

Assuming that *m* damage-related variables *x*_1_, *x*_2_, …, *x_m_* are selected for PCA, and that *n* sets of observations are available, the observed value of the *j*-th parameter in the *i*-th observation is denoted as *x_ij_*. To eliminate the influence of dimensional inconsistency and scale disparity among variables, each original data point *x_ij_* is standardized to obtain a dimensionless normalized value *x_ij_*.

(1) $${\tilde{x}}_{ij}=\frac{x_{ij}-{\bar{x}}_{j}}{s_{j}},\;(i=1,\;2,\;\cdots ,\;n;\;j=1,\;2,\;\cdots ,\;m)$$

In this context, ${\bar{x}}_{j}=\frac{1}{n}\sum_{i=1}^{n}x_{ij},\;s_{j}=\sqrt{\frac{1}{n-1}\sum_{i=1}^{n}{\left(x_{ij}-{\bar{x}}_{j}\right)}^{2}},\;(j=1\;,\;2,\;\cdots ,\;m)$. Here, ${\bar{x}}_{j}$ and $s_{j}$ denote the sample mean and standard deviation of the *j*-th variable, respectively. The standardized form of the damage-sensitive parameter is given by the following expression:

(2) $${\tilde{x}}_{i}=\frac{x_{i}-{\bar{x}}_{j}}{s_{i}},\;(i=1,\;2,\;\cdots ,m)$$

(2) Calculation of the correlation matrix R among variables

The correlation coefficient matrix R = (*r_ij_*)*_m_*_×*m*_ is constructed to quantify the linear relationships among the standardized variables. Each element *r_ii_* represents the Pearson correlation coefficient between the *i*-th and *j*-th variables, and is computed as follows:

(3) $$r_{ij}=\frac{\sum_{k=1}^{n}{\bar{x}}_{ki}\cdot {\bar{x}}_{kj}}{n-1},\;(i,\;j=1,\;2,\;\cdots ,\;m)$$

where *x*_*ki*_ and *x*_*kj*_ are the standardized values of the *i*-th and *j*-th variables in the *k*-th sample, and *n* is the total number of observations.

(3) Determination of eigenvalues and eigenvectors of the correlation matrix R

The correlation matrix R is then decomposed to obtain its eigenvalues *λ*_1_ ≥ *λ*_2_… ≥ *λ_m_* ≥ 0, and the corresponding eigenvectors *u*_1_, *u*_2_, ⋯, *u*_*m*_, where each eigenvector is defined as *u_j_* = (*u*_1*j*_, *u*_2*j*_, …, *u_nj_*)*^T^*. These eigenvectors are used as transformation axes to construct a new set of uncorrelated variables—namely, the principal components.

Each principal component is a linear combination of the original standardized variables, and together, they capture the maximum amount of variance in the original dataset with progressively decreasing explanatory power.

(4) $$\left\{\begin{array}{c} y_{1}=u_{11}{\bar{x}}_{1}+u_{21}{\bar{x}}_{2}+\cdots +u_{n1}{\bar{x}}_{n}\\ y_{2}=u_{12}{\bar{x}}_{1}+u_{22}{\bar{x}}_{2}+\cdots +u_{n2}{\bar{x}}_{n}\\ \vdots \\ y_{m}=u_{1m}{\bar{x}}_{1}+u_{2m}{\bar{x}}_{2}+\cdots +u_{nm}{\bar{x}}_{n} \end{array}\right.$$

(4) Construction of principal components

To compute the explained variance and cumulative variance of the eigenvalues λ_j_ (where j = 1, 2, ⋯, *m*), the explained variance ratio of the *j*-th principal component *y*_*j*_ is given by the following:

(5) $$b_{j}=\frac{\lambda_{j}}{\sum_{k=1}^{m}\lambda_{k}},\;(j=1,\;2,\;\cdots ,\;m)$$

The cumulative contribution rate of the principal components y_1_, y_2_, …, y_p_ is then calculated accordingly.

(6) $$\alpha_{p}=\frac{\sum_{k=1}^{p}\lambda_{k}}{\sum_{k=1}^{m}\lambda_{k}}$$

When *α_p_* approaches 1 (typically *α_p_* = 0.85, 0.90, 0.95), the first *p* principal components *y*_1_, *y*_2_, …, *y_p_* are selected to represent the original *m* damage-related variables.

(5) Computation of composite scores

(7) $$Z=\sum_{j=1}^{p}b_{j}y_{j}$$

where *b_j_* denotes the contribution rate of the *j*-th principal component.

**Figure 5:**
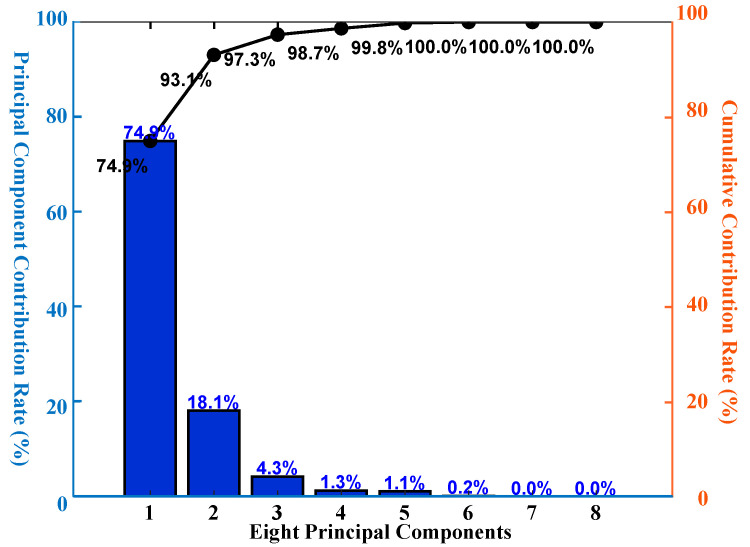
Eight principal components and their explained variance.

Figure 5 presents the contribution rate of each of the *p* principal components. To further investigate which components are most correlated with fatigue prediction, principal component loading analysis is introduced. In PCA, loadings indicate the projection of the original variables onto each principal component, thereby facilitating the physical interpretation of the components. The calculation is as follows:

(8) $$L=V\times \sqrt{E}$$

where *V* is the matrix of eigenvectors, and *E* is the diagonal matrix of corresponding eigenvalues.

**Figure 6:**
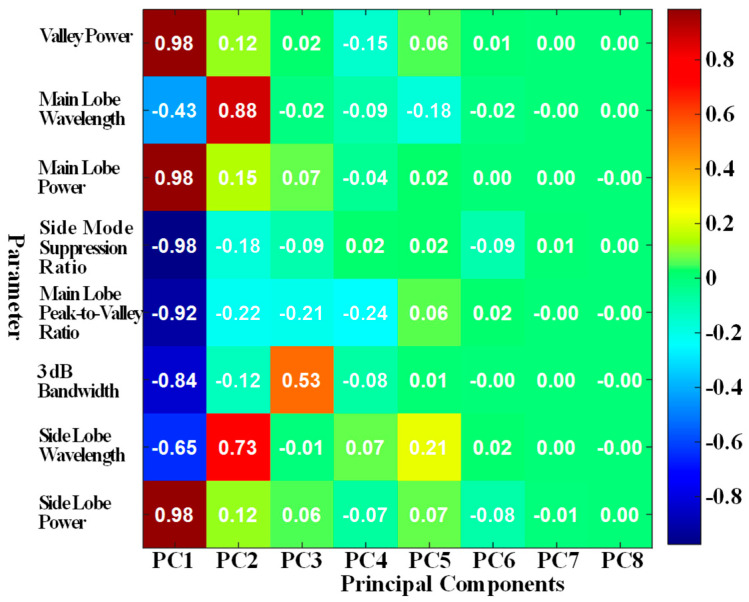
Heatmap of principal component loading matrix.

Figure 6 presents the heatmap of the principal component loading matrix. As shown, in the PC1 column, the main peak valley, main peak power, and side-lobe power exhibit the strongest positive correlations with the first principal component, indicating their significant contribution to this component. Conversely, the ratio of main peak power to side-lobe power and the ratio of main peak power to its valley value show the most substantial negative loadings, implying a strong inverse relationship with PC1.

As discussed, the ratio of main peak power to side-lobe power serves as a key indicator of spectral integrity and system stability, while the ratio of main peak power to its corresponding valley value reflects the sharpness and definition of the primary reflection peak. These two features are sensitive to changes in structural integrity and fatigue damage.

Based on their dominant roles in the PC1 structure and their clear physical significance, the first principal component is selected as the input feature for the fatigue life prediction model. Additionally, the ratio of main peak power to side-lobe power and the ratio of main peak power to its valley value are chosen as the primary damage-sensitive variables for fatigue state characterization and prognostics.

### 3.2. Normalization Methodology

When constructing the fatigue life model of the CFRP-FBG sensor, the experimental dataset includes feature parameters representing multiple physical quantities, which vary significantly in magnitude and characteristics. Directly using these features for modeling may cause those with larger numerical values to dominate, while features with smaller values are neglected, thereby adversely affecting the model training and prediction accuracy. Therefore, data normalization is essential. Common normalization methods include zero-mean standardization, min–max normalization, logarithmic transformation, and Box–Cox transformation. Among these, min–max normalization is suitable for scenarios where the data range is relatively fixed; it scales the data to the range of [0, 1] or [−1, 1], ensuring all features lie within the same numerical range. This improves the model’s stability and prediction accuracy while accelerating training convergence. Hence, this study adopts the min–max normalization method to scale the experimental data to the [0, 1] interval, enhancing model robustness and overall computational efficiency. The formula for min–max normalization is given as follows:

(9) $$Y=\frac{X-X_{\min}}{X_{\max}-X_{\min}}$$

where X and Y denote the values of a given parameter before and after normalization, respectively, and X_min_ and X_max_ represent the minimum and maximum values of that parameter within the same dimension of the dataset.

### 3.3. Evaluation Metrics for Prediction Performance

This study utilizes two deep learning architectures—namely, the Backpropagation (BP) Neural Network and the Long Short-Term Memory (LSTM) Neural Network—to predict the fatigue life of CFRP-FBG sensor-reinforced RC beams. Owing to the inherent differences in their predictive algorithms and performance characteristics, it is imperative to establish consistent and rigorous evaluation criteria to objectively assess the predictive efficacy of each model, thereby facilitating the selection of the most suitable fatigue life prediction framework.

To this end, Mean Squared Error (MSE), Root Mean Squared Error (RMSE), and Mean Absolute Error (MAE) are employed as quantitative metrics to comprehensively evaluate and compare the prediction accuracy of the models.

MSE quantifies the average of the squared discrepancies between predicted values and corresponding ground truth observations. By computing the mean of the squared residuals, MSE effectively captures the variance of prediction errors. A smaller MSE value signifies superior model fitting and predictive performance. The mathematical expression for MSE is defined as follows:

(10) $$\mathrm{MSE}=\frac{1}{n}\sum_{i=1}^{n}{\left(y_{i}-{\hat{y}}_{i}\right)}^{2}$$

Here, *n* denotes the total number of samples, $y_{i}$ represents the true (observed) value, and ${\hat{y}}_{i}$ denotes the corresponding predicted value.

Root Mean Squared Error (RMSE) is used to assess the deviation between predicted values and actual observations. It is calculated by taking the square root of the mean of squared errors, thereby reflecting the overall magnitude of prediction error. A lower RMSE indicates better model performance in terms of fit. The mathematical formulation of RMSE is as follows:

(11) $$RMSE=\sqrt{MSE}$$

Mean Absolute Error (MAE) is a widely used metric for evaluating the prediction accuracy of regression models on continuous data. It measures the average absolute difference between predicted values and true values, thereby quantifying the average magnitude of prediction error. A smaller MAE value signifies higher prediction accuracy. Notably, the unit of MAE is consistent with that of the original data, which enhances interpretability. The MAE is computed as follows:

(12) $$\mathrm{MAE}=\frac{1}{n}\sum_{i=1}^{n}\left|y_{i}-{\hat{y}}_{i}\right|$$

## 4. Construction of Predictive Models and Prognostic Evaluation

### 4.1. Benchmark Neural Network Model Based on BP

To evaluate the predictive performance of the proposed LSTM neural network in modeling the fatigue life of CFRP-FBG sensor-reinforced RC beams, a conventional three-layer feedforward BP neural network is employed as a baseline for comparative analysis. A structured experiment is designed to assess the capability of both models in handling high-dimensional fatigue feature sequences. The dataset is partitioned into training and testing sets using stratified random sampling with an 80:20 ratio, ensuring effective parameter learning and a robust evaluation of generalization performance.

**Figure 7:**
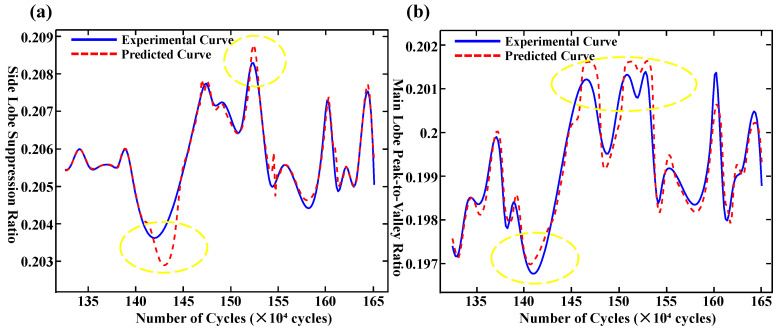
(**a**) Fitting results of side-mode suppression ratio using BP neural network. (**b**) Fitting results of main-lobe peak-to-valley ratio using BP neural network.

Figure 7a,b depicts the fitting results of the BP neural network for the side-mode suppression ratio and the main-lobe peak-to-valley ratio, respectively. Within the fatigue cycle range of 1.32 million to 1.40 million, the predicted values exhibit general agreement with the measured data, indicating the model’s ability to capture short-term spectral variation trends. However, as the fatigue cycles progress, the model performance deteriorates significantly, especially at critical inflection points, such as at 1.43 million and 1.52 million cycles in Figure 7a, and at 1.41 million, 1.48 million, and 1.53 million cycles in Figure 7b, highlighting the model’s limited capacity to represent non-stationary and long-term dependencies.

Quantitative error analysis further corroborates these findings. The Root Mean Squared Error (RMSE), Mean Absolute Error (MAE), and Mean Squared Error (MSE) for the side-mode suppression ratio are 0.2487, 0.1346, and 0.0680, respectively; for the main-lobe peak-to-valley ratio, the corresponding values are 0.2698, 0.2158, and 0.0728. While the model demonstrates acceptable fitting accuracy during the initial steady-state stage, its overall predictive precision remains suboptimal, indicating inherent limitations in generalization capacity and robustness.

**Figure 8:**
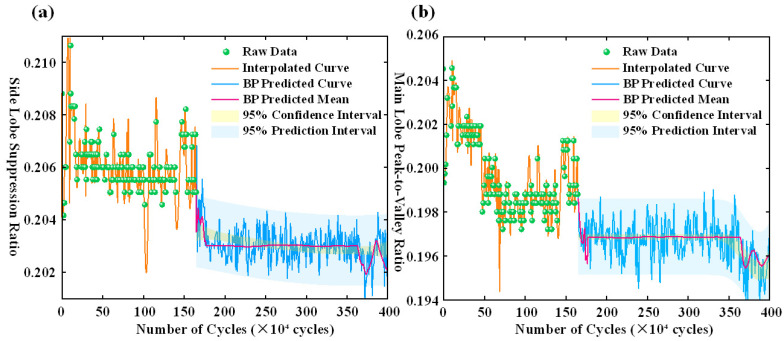
(**a**) Predicted side-mode suppression ratio across fatigue cycles using BP neural network. (**b**) Predicted main-lobe peak-to-valley ratio across fatigue cycles using BP neural network.

Figure 8 provides a more comprehensive view of the BP network’s predictive performance across the full fatigue cycle. In the early stage (0–0.5 million cycles), the model succeeds in approximately tracking the evolution of key spectral features. However, from 2.5 million cycles onward, the predicted values begin to oscillate markedly, accompanied by a significant expansion in confidence intervals. This suggests that the BP model is highly susceptible to nonlinear disturbances and trend drift, resulting in diminished predictive stability. In the later stage (3.6–4.0 million cycles), the model exhibits erratic behavior, underscoring a pronounced deficiency in long-term temporal generalization and stability.

In summary, although the BP neural network demonstrates moderate performance in low-cycle, weakly nonlinear fatigue prediction scenarios, its lack of temporal memory and inadequate capacity for modeling complex degradation dynamics severely constrain its applicability to long-sequence, strongly non-stationary fatigue processes. These limitations necessitate the adoption of advanced deep learning architectures—such as LSTM networks—that are inherently equipped with temporal gating mechanisms and are better suited for capturing long-range dependencies and multi-phase fatigue degradation patterns.

### 4.2. Structural Design and Optimization Methodology for the LSTM Neural Network

Building upon the findings from the previous evaluation of the BP neural network, it has been observed that this conventional architecture exhibits notable deficiencies when applied to the nonlinear and non-stationary time-series data derived from CFRP-FBG sensors for fatigue life prediction. Specifically, its limitations are manifested in suboptimal fitting accuracy, inadequate modeling of long-term dependencies, and a pronounced tendency toward overfitting in complex degradation scenarios.

**Figure 9:**
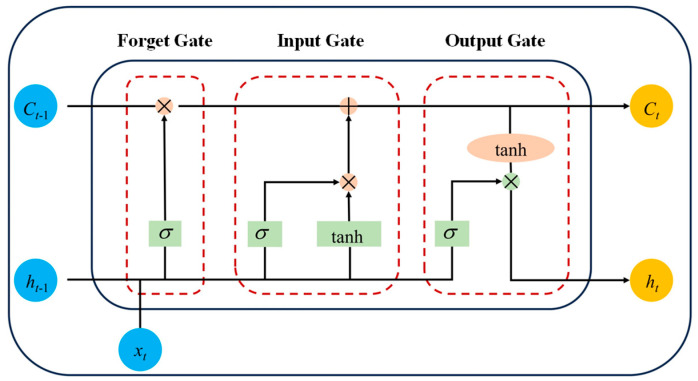
Structural schematic of an LSTM unit.

To address these shortcomings and enhance the model’s representational capacity for capturing the intricate dynamics of fatigue evolution, this study adopts the Long Short-Term Memory (LSTM) neural network as the principal modeling framework [20,21]. The LSTM architecture extends the conventional Recurrent Neural Network (RNN) by introducing memory cells and a gating mechanism, which collectively regulates the flow of information through controlled processes of retention, update, and forgetting [22,23,24]. These features endow the model with the ability to preserve salient temporal information over extended sequences, thereby facilitating the effective learning of long-range dependencies [25,26]. A schematic illustration of the internal structure of an LSTM unit is provided in Figure 9.

The LSTM architecture comprises three integral gating mechanisms: the forget gate, input gate, and output gate [20,27]. The forget gate selectively determines which information to expunge from the cell state at each time step, informed by the current input and the preceding hidden state, thus preventing the accumulation of irrelevant or redundant data. Concurrently, the input gate regulates the incorporation of new candidate information into the cell state, jointly orchestrating the update of the internal memory representation. The output gate governs the exposure of the processed cell state, filtering and transmitting the relevant information to subsequent time steps.

These gating operations afford the LSTM model a robust mechanism for controlling the flow of gradients during Backpropagation through time, effectively mitigating the vanishing and exploding gradient problems inherent in conventional recurrent neural networks. Consequently, LSTM networks maintain stable gradient propagation over extended temporal dependencies, enabling accurate modeling of long-range sequential data and complex temporal dynamics.

To further augment the predictive capability of the LSTM model, this study incorporates two pivotal optimization strategies:

(1) Dropout Regularization

**Figure 10:**
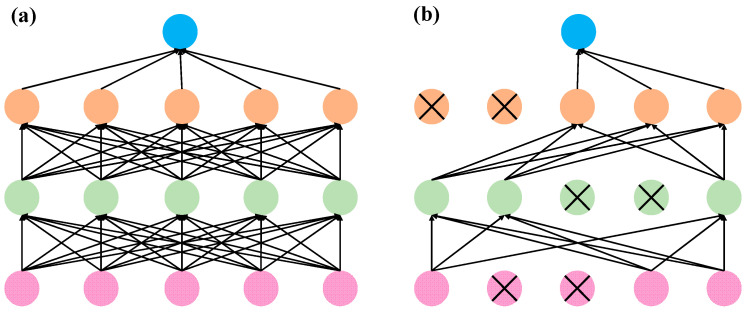
Comparison between models trained with and without Dropout (**a**) During training; (**b**) During inference.

Deep learning models with a large number of parameters are prone to overfitting, particularly when the available training samples are limited. Overfitting results in excellent performance on the training set but poor generalization on unseen data. To alleviate this issue, Hinton et al. proposed the Dropout technique, a regularization method that randomly deactivates a fraction of neurons during training to reduce co-adaptation among neurons and improve the model’s generalization capability [28]. As illustrated in Figure 10, the application of Dropout substantially enhances model robustness.

During training, Dropout stochastically ignores the outputs of neurons with a dropout probability *p*. The dropped neurons neither participate in forward propagation nor in gradient updates, effectively creating a different “thinned” subnetwork in each training iteration that continues with normal forward and backward passes. This stochastic network sampling forces the model to learn more robust features, as it cannot rely on any single neuron’s presence.

During inference, all neurons are activated to leverage the full representational capacity of the network. To reconcile the discrepancy in activation magnitudes between training and testing, the neuron outputs are scaled by the retention probability *p*, ensuring consistency in the expected output.

Collectively, Dropout functions as a robust regularization mechanism, effectively reducing overfitting, improving model generalizability, and concurrently decreasing computational overhead during training. This technique substantially bolsters the LSTM model’s robustness and its capacity to generalize to novel, unseen data.

(2) Adam Optimization Algorithm

In the field of deep learning, the choice of optimization algorithms significantly impacts the convergence speed of model training and the ultimate performance. To effectively minimize the loss function, appropriate optimization methods are essential. Commonly used optimization algorithms include gradient descent, stochastic gradient descent, Momentum, Adagrad, and Adam. Among these, the Adam algorithm, proposed by Kingma et al., integrates the advantages of Momentum and RMSprop, demonstrating strong capabilities in optimizing non-stationary objective functions, which has led to its widespread adoption in deep learning model training [29]. Adam adaptively estimates the first and second moments of gradients to dynamically adjust the learning rates for each parameter, thereby enhancing the stability and efficiency of the optimization process. To further highlight the advantages of Adam and its variants in improving model performance and training efficiency, numerous studies have proposed various enhancements and extensions.

For instance, HN_Adam, an improved version of Adam, introduces automatic adjustment of parameter update steps and incorporates the AMSGrad mechanism, resulting in significant improvements in convergence speed and generalization capability. Extensive experimental results demonstrate that HN_Adam outperforms the original Adam and other advanced adaptive optimization algorithms, such as AdaBelief, in terms of test accuracy and convergence efficiency on benchmark datasets like MNIST and CIFAR-10 [30].

Moreover, in large-scale distributed machine learning tasks, a novel federated learning algorithm that integrates a lazy update strategy into a distributed Adam framework has been proposed. This approach unifies learning rates and reduces communication overheads, substantially enhancing training efficiency and model performance. Experimental evaluations on the CIFAR-10 dataset indicate that this method surpasses traditional federated averaging algorithms and their momentum-based variants [31].

These examples collectively illustrate the superior performance of Adam and its variants in deep learning optimization, further validating their effectiveness in accelerating convergence and improving generalization.

Specifically, Adam updates parameters using the following equations:

(a) First-Order Moment Estimation

(13) $$m_{t}=\beta_{1}m_{t-1}+(1-\beta_{1})g_{t}$$

(b) Second-Order Moment Estimation

(14) $$v_{t}=\beta_{2}v_{t-1}+(1-\beta_{2})g_{t}^{2}$$

(c) Bias-Corrected Estimates

(15) $${\hat{m}}_{t}=\frac{m_{t}}{1-\beta_{1}^{t}},\;{\hat{v}}_{t}=\frac{v_{t}}{1-\beta_{2}^{t}}$$

(d) Parameter Update Rule

(16) $$\theta_{t}=\theta_{t-1}-\frac{\alpha }{\sqrt{{\hat{v}}_{t}}+\epsilon }{\hat{m}}_{t}$$

In these equations, *m_t_* and *v_t_* denote the biased estimates of the first-order (mean) and second-order (uncentered variance) moments of the gradients, respectively. The coefficients *β_1_* and *β_2_* represent the exponential decay rates for the moving averages of the gradients and their squares. The scalar *α* is the initial learning rate, while *ε* is a small constant added to improve numerical stability and prevent division by zero. *θ_t_* denotes the model parameters at iteration *t*, and *g_t_* represents the gradient of the loss function with respect to the parameters at the same iteration.

(3) Model Hyperparameter Configuration

The maximum number of iterations *m* defines the upper bound on the total number of training steps. Once this threshold is reached, the training process is forcibly terminated to prevent unnecessary computation. The maximum number of epochs *e* represents the total number of complete passes through the training dataset. Increasing *e* can improve model accuracy by allowing further convergence, but excessive values may lead to overfitting. The incorporation of a Dropout layer with a dropout rate *D* = 0.2 is employed to mitigate overfitting. This mechanism randomly disables 20% of the neurons during each training iteration, thereby enhancing the model’s generalization capability. The gradient clipping threshold *G* is introduced to prevent gradient explosion, a phenomenon particularly common in deep neural networks. If the computed gradient exceeds this threshold, it is rescaled to ensure numerical stability during parameter updates. The initial learning rate *R* governs the magnitude of each parameter update. While smaller learning rates promote stable convergence, they typically require more iterations to reach an optimal solution. To further enhance training efficiency and convergence accuracy, a learning rate scheduling strategy *S* is adopted. This strategy adaptively reduces the learning rate at predetermined epochs, allowing for finer optimization in later stages and reducing the risk of missing the global optimum. Lastly, the training verbosity level *V* determines the granularity of log output during training. When *V* = 0, only essential training metrics are displayed, minimizing console clutter. When *V* = 1, detailed logs such as loss values per epoch are provided, facilitating real-time monitoring and diagnostic analysis of the training process. The parameter settings of LSTM neural network are shown in Table 2.

### 4.3. Prediction Results and Performance Evaluation of the LSTM Model

**Figure 11:**
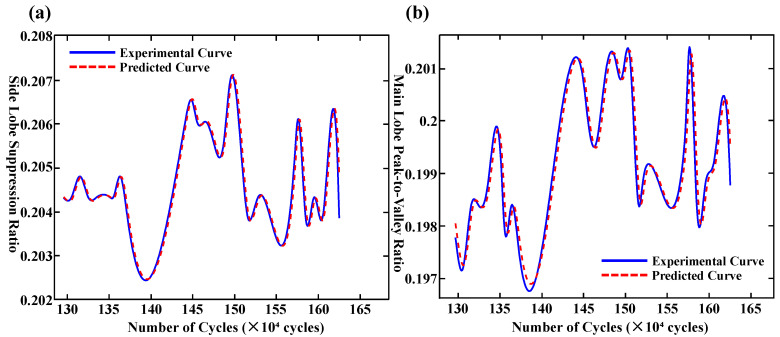
(**a**) Curve fitting of side-mode suppression ratio. (**b**) Curve fitting of main-lobe peak-to-valley ratio.

Figure 11a,b illustrate the predictive performance of the LSTM neural network on two representative datasets. In both subplots, the blue solid line represents the measured values, while the red dashed line corresponds to the predicted outputs generated by the model. As shown in Figure 11a, the time series exhibits significant volatility, characterized by multiple distinct peaks and valleys. The LSTM model demonstrates excellent performance in capturing these complex patterns, particularly within the X-axis interval of 130–165, where the predicted trajectory closely aligns with the observed data. Although slight deviations occur near several peak regions, the model exhibits a strong capability for learning nonlinear dynamic behaviors. Overall, the fitting performance is considered highly satisfactory. In contrast, the time series presented in Figure 11b displays relatively lower amplitude fluctuations, though notable trend shifts remain evident. The LSTM model successfully captures the overall trend and reflects the temporal dynamics inherent in the data. While minor deviations are observed near X = 138, the model maintains a stable prediction performance across most of the sequence. From a quantitative standpoint, the RMSE, MAE, and MSE for Figure 11a are 0.1616, 0.1150, and 0.0261, respectively. For Figure 11b, the corresponding values are 0.2126, 0.1620, and 0.0452. These performance metrics collectively indicate that the LSTM model achieves a high level of predictive accuracy for both fatigue-sensitive parameters, effectively capturing critical features and underlying temporal patterns in the dataset.

**Figure 12:**
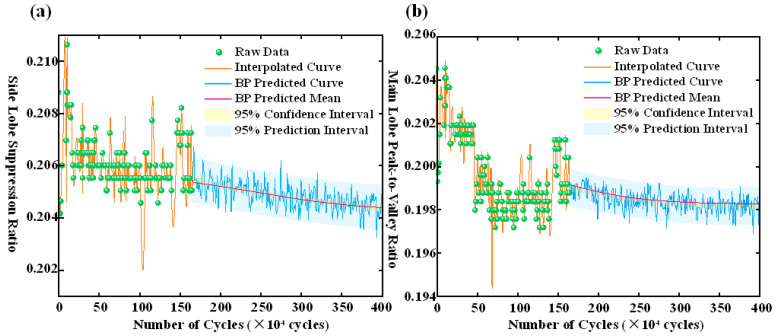
(**a**) Prediction curve of side-mode suppression ratio. (**b**) Prediction curve of main-lobe peak-to-valley ratio.

Figure 12a,b, respectively, present the experimental data and LSTM prediction results of the side-mode suppression ratio and the main-lobe peak-to-valley ratio as functions of fatigue cycle count. Green scatter points represent experimental measurements; the orange solid line denotes the interpolation of experimental data; the blue solid line corresponds to the LSTM prediction; the pink solid line indicates the predicted mean; the dark blue shaded area represents the 95% confidence interval; and the light blue shaded area depicts the 95% prediction interval. Figure 12a shows that the side-mode suppression ratio fluctuates markedly within the first 0 to 500,000 cycles, and then stabilizes. The LSTM prediction curve effectively captures the overall degradation trend, with a relatively narrow confidence interval prior to 2.5 million cycles, indicating low prediction uncertainty and high fitting accuracy. Compared to the BP neural network, the LSTM maintains superior stability beyond 2.5 million cycles, with significantly reduced prediction volatility and confidence interval expansion, demonstrating enhanced generalization capability. In Figure 12b, the main-lobe peak-to-valley ratio exhibits a pronounced initial decline followed by stabilization. The LSTM prediction curve aligns closely with experimental data across the entire cycle range, showing reduced volatility and narrower confidence intervals after 2.5 million cycles, further confirming its superior stability for long-term prediction. Notably, the LSTM predictions for both metrics display no abnormal abrupt drops or rebounds, maintaining smooth overall trends, which demonstrates the model’s capability to accurately characterize the long-term fatigue degradation process.

Table 3 and Table 4 summarize the normalized error metrics for the predictions of the side-mode suppression ratio and main-lobe peak-to-valley ratio, respectively. The LSTM neural network outperforms the traditional BP neural network across all error evaluation metrics, with the extent of improvement varying slightly according to the specific task characteristics. For the side-mode suppression ratio prediction, the LSTM demonstrates a marked advantage in controlling extreme errors, with the MSE reduced by 61.62% and RMSE decreased by 34.99% compared to the BP network. This indicates a substantial enhancement in the model’s robustness against outliers and overall prediction stability. In the main-lobe peak-to-valley ratio prediction, the LSTM is particularly effective in suppressing local errors, achieving a 24.9% reduction in MAE. However, the overall RMSE reduction of 21.2% is slightly lower than that observed for the side-mode suppression ratio, reflecting the inherent volatility of this metric, which poses additional challenges to prediction accuracy.

In conclusion, the LSTM neural network exhibits marked superiority in the time-series forecasting of key fatigue-sensitive parameters. Its pronounced capabilities in mitigating extreme errors, stabilizing overall predictive performance, and refining local error dynamics culminate in a significantly improved congruence with experimental observations and tighter confidence intervals. These strengths underscore the model’s robustness in accurately characterizing long-term degradation trajectories and its exceptional generalization across complex fatigue behaviors. Consequently, the LSTM framework establishes a powerful and reliable foundation for advanced fatigue life prediction in intricate structural systems, facilitating enhanced prognostic accuracy and structural health management.

## 5. Conclusions

This study systematically addresses the lifetime prediction of CFRP-FBG sensors subjected to complex fatigue loading by developing an advanced LSTM-based time-series modeling framework. Fatigue experiments were performed on reinforced concrete beams instrumented with CFRP-FBG sensors, capturing multiple key spectral features including side-mode suppression ratio and main-lobe peak-to-valley ratio. Principal Component Analysis (PCA) was employed to reduce data dimensionality and extract these two principal indicators closely correlated with sensor degradation and structural fatigue behavior.

To effectively model the nonlinear and long-term temporal dependencies of the sensor data, an LSTM network architecture was constructed, incorporating the Adam optimizer for adaptive learning rate adjustment and Dropout layers to prevent overfitting. Input features were normalized using min–max scaling to stabilize model training and improve convergence. The model’s performance was benchmarked against a conventional Backpropagation (BP) neural network.

Quantitative evaluation shows that the LSTM significantly outperforms the BP network in both predictive accuracy and robustness. Specifically, for the side-mode suppression ratio, LSTM reduced the mean squared error (MSE) by 61.62% and the root mean squared error (RMSE) by 34.99%, indicating enhanced capability to mitigate outliers and noisy fluctuations. For the main-lobe peak-to-valley ratio, LSTM decreased the mean absolute error (MAE) by 24.9% and RMSE by 21.2%, demonstrating improved sensitivity to subtle local variations inherent in fatigue progression.

Temporal trend analysis coupled with confidence interval estimation reveals that the LSTM model reliably captures the fatigue-induced degradation dynamics with narrow confidence bands and low prediction variance. Importantly, during extended fatigue cycling exceeding one million cycles, the LSTM maintains prediction stability without the erratic oscillations or anomalous rebounds commonly observed in traditional models. This robustness is attributed to the LSTM’s gated recurrent units effectively modeling long-term dependencies and filtering noise.

In conclusion, the proposed LSTM-based time-series prediction methodology not only advances the precision and stability of CFRP-FBG sensor lifetime assessments but also enhances the handling of extreme deviations and localized fluctuations. Its superior nonlinear representation and temporal dependency learning capabilities establish a reliable and intelligent framework for real-time sensor state diagnostics and predictive maintenance in structural health monitoring systems. Future implementations of this approach are expected to facilitate continuous, large-scale monitoring of critical infrastructures, providing data-driven insights to support safety assurance and optimized maintenance decision making, thereby accelerating the integration of intelligent sensing technologies within structural engineering practice.

## Figures and Tables

**Table 1:** Performance parameters of FBG sensors.

Parameter	Specification
Central Wavelength (nm)	1530–1570
Grating Length (mm)	10
Reflectivity	≥80%
Fiber Core Diameter (µm)	8
Cladding Diameter (µm)	125
Fiber Outer Diameter (µm)	250
Heat Treatment Temperature (°C)	130

**Table 2:** LSTM neural network hyperparameter configuration.

Parameter	m	D	e	G	R	V
Value	1000	0.2	500	1	0.001	0 or 1

**Table 3:** Normalized errors of side-mode suppression ratio.

Metric	BP Neural Network	LSTM Neural Network	Change Trend
RMSE	0.2487	0.1616	−34.99%
MAE	0.1346	0.1150	−14.6%
MSE	0.068	0.0261	−61.62%

**Table 4:** Normalized errors of main-lobe peak-to-valley ratio.

Metric	BP Neural Network	LSTM Neural Network	Change Trend
RMSE	0.2698	0.2126	−21.2%
MAE	0.2158	0.1620	−24.9%
MSE	0.0728	0.0452	−37.9%

## Data Availability

The original contributions presented in this study are included in the article. Further inquiries can be directed to the corresponding authors.
